# The association between coronary artery calcification and vectorcardiography in mechanically ventilated COVID-19 patients: the Maastricht Intensive Care COVID cohort

**DOI:** 10.1186/s40635-024-00611-0

**Published:** 2024-03-07

**Authors:** Eda Aydeniz, Frank van Rosmalen, Jip de Kok, Bibi Martens, Alma M. A. Mingels, Mustafa Emin Canakci, Casper Mihl, Kevin Vernooy, Frits W. Prinzen, Joachim E. Wildberger, Iwan C. C. van der Horst, Bas C. T. van Bussel, Rob G. H. Driessen

**Affiliations:** 1 Department of Intensive Care Medicine, Maastricht University Medical Center+, Maastricht, The Netherlands; 2 Cardiovascular Research Institute Maastricht (CARIM), Maastricht University, Maastricht, The Netherlands; 3 Department of Radiology and Nuclear Medicine, Maastricht University Medical Center+, Maastricht, The Netherlands; 4 Central Diagnostic Laboratory, Maastricht University Medical Center+, Maastricht, The Netherlands; 5 Emergency Department, Eskisehir Osmangazi University School of Medicine, Eskisehir, Turkey; 6 Department of Cardiology, Maastricht University Medical Center +, Maastricht, The Netherlands; 7 Department of Physiology, Maastricht University, Maastricht, The Netherlands; 8 Care and Public Health Research Institute (CAPHRI), Maastricht University, Maastricht, The Netherlands

**Keywords:** Vectorcardiography, Vascular calcification, Intensive care unit, COVID-19

## Abstract

**Background:**

Coronary artery calcification (CAC) is associated with poor outcome in critically ill patients. A deterioration in cardiac conduction and loss of myocardial tissue could be an underlying cause. Vectorcardiography (VCG) and cardiac biomarkers provide insight into these underlying causes. The aim of this study was to investigate whether a high degree of CAC is associated with VCG-derived variables and biomarkers, including high-sensitivity troponin-T (hs-cTnT) and N-terminal pro-B-type natriuretic peptide (NT-proBNP).

**Methods:**

Mechanically ventilated coronavirus-19 (COVID-19) patients with an available chest computed tomography (CT) and 12-lead electrocardiogram (ECG) were studied. CAC scores were determined using chest CT scans. Patients were categorized into 3 sex-specific tertiles: low, intermediate, and high CAC. Daily 12 leads-ECGs were converted to VCGs. Daily hs-cTnT and NT-proBNP levels were determined. Linear mixed-effects regression models examined the associations between CAC tertiles and VCG variables, and between CAC tertiles and hs-cTnT or NT-proBNP levels.

**Results:**

In this study, 205 patients (73.2% men, median age 65 years [IQR 57.0; 71.0]) were included. Compared to the lowest CAC tertile, the highest CAC tertile had a larger QRS area at baseline (6.65 µVs larger [1.50; 11.81], *p* = 0.012), which decreased during admission (− 0.27 µVs per day [− 0.43; − 0.11], *p* = 0.001). Patients with the highest CAC tertile also had a longer QRS duration (12.02 ms longer [4.74; 19.30], *p* = 0.001), higher levels of log hs-cTnT (0.79 ng/L higher [0.40; 1.19], *p* < 0.001) and log NT-proBNP (0.83 pmol/L higher [0.30; 1.37], *p* = 0.002).

**Conclusion:**

Patients with a high degree of CAC had the largest QRS area and higher QRS amplitude, which decreased more over time when compared to patients with a low degree of CAC. These results suggest that CAC might contribute to loss of myocardial tissue during critical illness. These insights could improve risk stratification and prognostication of patients with critical illness.

**Supplementary Information:**

The online version contains supplementary material available at 10.1186/s40635-024-00611-0.

## Background

Coronavirus 19 (COVID-19) is an infectious disease caused by severe acute respiratory syndrome coronavirus 2 (SARS-CoV-2) that has shown the potential to affect various organ systems, including the cardiovascular system [1]. In several studies, it has been shown that myocardial injury was associated with increased mortality in hospitalized COVID-19 patients [2–4]. Biomarkers, including high-sensitivity troponin-T (hs-cTnT), creatine kinase-MB (CK-MB) fraction, myoglobin, and N-terminal pro-B-type natriuretic peptide (NT-proBNP), have been found to be increased in COVID-19 patients with severe disease and are associated with adverse outcomes, including mortality [3, 5–8]. Moreover, a higher degree of coronary artery calcification (CAC), a marker for coronary atherosclerosis, is associated with more severe organ failure and worse outcomes in COVID-19 patients [9–12].

In several studies, abnormal electrocardiographic (ECG) findings were observed in COVID-19 patients, including ST segment changes, T wave inversion, and PR interval, QRS duration, and QT interval alterations [3, 13–15]. Vectorcardiography (VCG) has potential value for gaining further insight into cardiac disease. VCG provides a three-dimensional representation of the electrical activity of the heart, offering information about the direction and magnitude of electrical forces [16, 17]. Different parameters, including QRS-area, could be determined using this tool. The QRS area consists of QRS amplitude and duration. In previous studies, it was shown that VCG is a promising tool to improve patient selection for cardiac resynchronization therapy [17]. Although current studies do not specifically address the relationship between VCG and COVID-19, they emphasize the importance of monitoring heart health and cardiac injury biomarkers in COVID-19 patients [3, 17–19].

Whether patients with more severe coronary atherosclerosis face worse outcomes due to impaired cardiac conduction (i.e., slower conduction), and/or loss of myocardial tissue over time is unknown. Therefore, the aim of this study was to examine whether a high degree of CAC is associated with VCG-derived variables during admission for a critical illness. More specifically, we investigated whether patients with a higher degree of CAC have a decrease in QRS area, which consist of QRS duration and amplitude. Additionally, we investigated whether patients with a higher degree of CAC have elevated hs-cTnT and NT-proBNP. By analyzing VCG data, it may be possible to detect subtle changes in electrical activity, identify arrhythmogenic substrates, and assess the impact of COVID-19 on myocardial tissue and electrical properties. Such knowledge could improve risk stratification, prognostication, and management of COVID-19 patients with cardiac involvement.

## Methods

### Study design

The Maastricht Intensive Care COVID (MaastrICCht) cohort is a prospective observational cohort study described extensively elsewhere [20]. In short, this study was conducted in the Intensive Care Unit (ICU) of the Maastricht University Medical Center + (MUMC +), a tertiary hospital in the Netherlands. The study was approved by the medical ethics committee (METC) of MUMC + (2020–1565/3 00 523), which was based on the Declaration of Helsinki. Moreover, this study was registered in the International Clinical Trials Registry Platform (NL8613; 12/05/2020). This manuscript was written according to the "*Strengthening the Reporting of Observational Studies in Epidemiology*" (STROBE) guideline [21].

### Imaging population and Coronary Calcium Score

All mechanically ventilated patients admitted from March 2020 until October 2021 with a chest computed tomography (CT) scan highly suggestive of COVID-19, as indicated by a score of 4 or 5 on the COVID-19 Reporting and Data System (CO-RADS) and/or a polymerase chain reaction (PCR) test positive for COVID-19, were included in this study. Imaging was conducted on 4 different scanners within the cohort, including a mobile scan unit (Alliance Medical equipped with Lightspeed 16, GE Healthcare, Milwaukee, WI) for clinically stable triage patients. Other available scanners were either used for unstable patients in the emergency department (SOMATOM Definition Flash, Siemens Healthineers, Forchheim, Germany) or for clinical inpatients (SOMATOM Force; SOMATOM Definition AS; Siemens Healthineers, respectively), with tube voltages between 90 and Sn150 kV.

CAC was graded on the data available on the PACS workstation (IMPAX, version 6.6.1.5003; AGFA HealthCare N.V., Mortsel, Belgium). The calcifications were assessed according to their location in the left main, left anterior descending, left circumflex artery, and right coronary artery, using a semiquantitative grading system, which has been described extensively elsewhere [22–26]. Calcifications were scored as absent (0), mild (1), moderate (2), or severe (3) for each coronary artery. The four separate scores were summed up to an overall grade varying between 0 and 12, where 0 indicates no CAC, and 12 indicates extensive coronary artery disease.

### Markers of myocardial injury and wall stress longitudinally during admission

Data on various clinical variables and biomarkers were collected daily, including hs-cTnT and NT-proBNP levels.

ECGs were performed daily, starting at intubation until either death or discharge from ICU. Twelve-lead ECGs were taken during the morning, at 25 mm/s and 10 mm/mV, and stored (MUSE Cardiology, GE Medical System). Post-processing was done on all ECGs that were converted into orthogonal VCG leads (X, Y, Z) using the Kors conversion matrix in MATLAB (MathWorks Inc.) [27]. After conversion, the start and end of the QRS complex and T-waves were manually determined. The electrical activation sequence of the ventricles was constructed, and the VCG variables were automatically calculated and extracted using MATLAB. VCG and ECG markers include QRS area, amplitude and duration. The QRS area was calculated as the integral under the QRS complex in the orthogonal leads based on the amplitude and duration. In addition, QRST area, T area, and QTc interval were determined. The QRST area was defined as the sum of the QRS area and the T area. The T area was similarly calculated as the integral under the T wave in the orthogonal leads.

Serum markers, including hs-cTnT (ng/L) and NT-proBNP (pmol/L), were assessed daily during admission using a Cobas 8000 analyzer (Roche Diagnostics, Mannheim, Germany). Assay characteristics were all according to the package inserts. For hs-cTnT, the limit of quantification was 3.0 ng/L at a coefficient of variation of 10% and an overall 99th percentile upper reference limit of 14 ng/L. For NT-proBNP, a concentration < 35 pmol/L was considered normal.

### Statistical analysis

Patient characteristics were categorized into sex-specific CAC tertiles, as previous studies suggest that CAC scores were higher in men compared to women and women were admitted less often due to COVID-19 [28–30]. This methods decreases the risk of bias by sex. Patients were categorized into tertile 1 (low CAC; reference), tertile 2 (intermediate CAC), and tertile 3 (high CAC). Variables are described as median and interquartile range (IQR), mean and SD, or as numbers and percentages, as appropriate. Baseline characteristics were compared using the Kruskal–Wallis test, one-way ANOVA, Fisher’s exact test, or chi-square test.

First, linear mixed-effects regression with a random intercept for time since intubation was used to investigate the longitudinal associations between CAC tertiles and the development of VCG variables over time. The models were adjusted for potential confounders, including age, sex, Acute Physiology And Chronic Health Evaluation score (APACHE-II), chronic lung disease, and liver conditions [9, 28, 30–35]. The primary models investigated whether CAC tertiles had an average difference over time in the serial variables under investigation. Moreover, the models were adjusted for serum markers, including hs-cTnT and NT-proBNP levels, to investigate whether the differences between CAC tertiles were affected by serum markers. Additionally, time (in days) and the interaction between time and CAC tertiles were added to the models to investigate the effect modification of the association between CAC and the variables under study by time (a statistically significant interaction term indicates that the trajectory in the VCG variable for the CAC tertile under investigation develops differently over time compared to the reference CAC tertile). The data were re-analyzed using CAC as a continuous variable to analyze the sensitivity of the models.

In addition, using similar models, linear mixed-effects regression with a random intercept for time since intubation was used to investigate the longitudinal associations between CAC tertiles and the development of serum markers, including hs-cTnT and NT-proBNP levels. Serum markers were log-transformed in order to meet the normality criteria. Statistical analyses were conducted using R version 4.1.2 (R Foundation for Statistical Computing, Vienna, Austria). A *p*-value < 0.05 and a *p*-value for interaction < 0.10 were considered statistically significant.

## Results

### Patient population

**Fig. 1:**
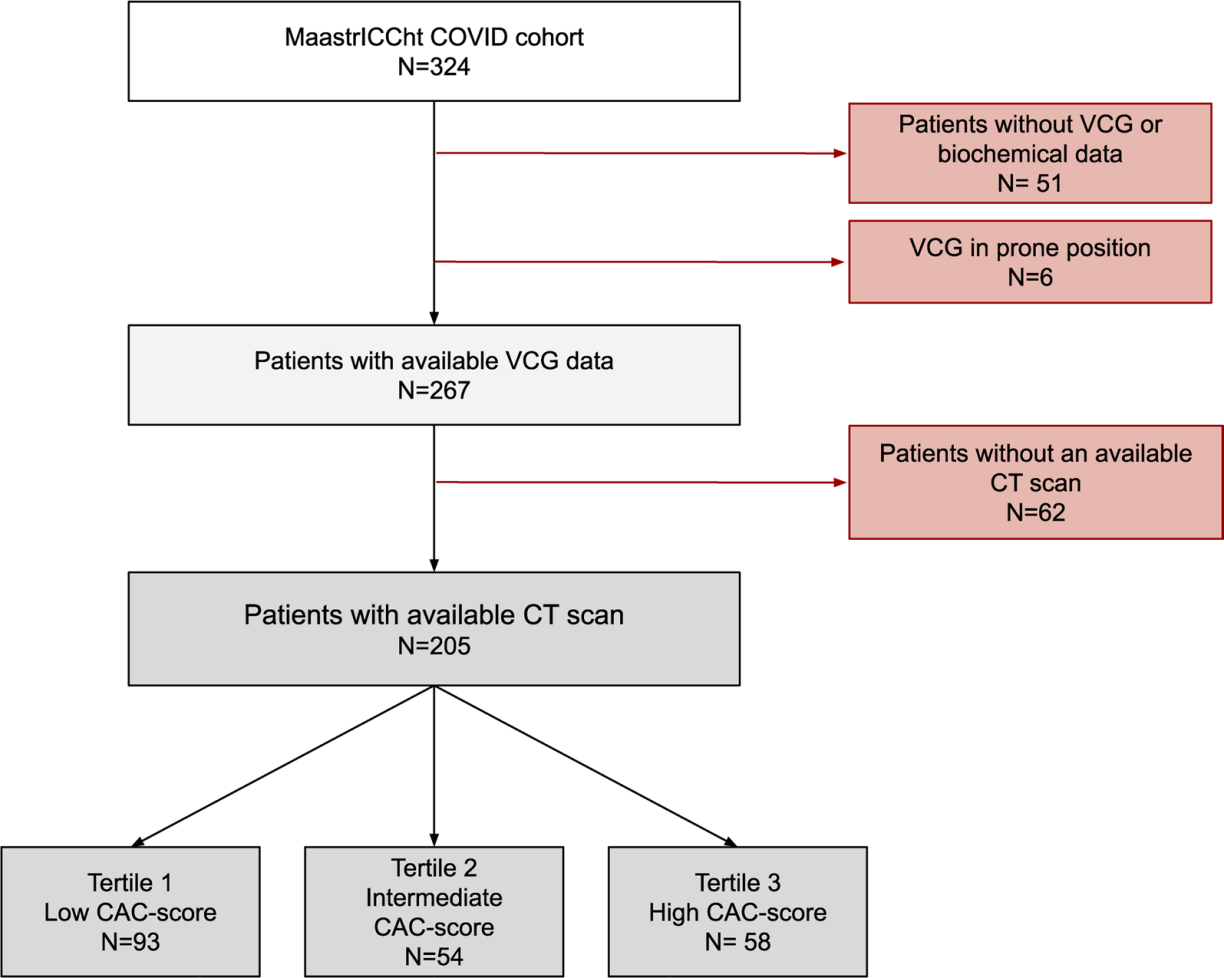
Flow diagram of the study population. Patients were enrolled in the Maastricht Intensive Care (MaastrICCht) COVID cohort. Patients without vectorcardiography (VCG), chest computed tomography (CT) scan, and biochemical data or with VCG measurements in the prone position were excluded from this study. CAC: coronary artery calcification

The MaastrICCht cohort comprised 324 mechanically ventilated COVID-19 patients, of whom 205 had undergone a chest CT scan with both ECG/VCG and serum biomarker data (Fig. 1). The total included patient population consisted of these 205 patients, of whom 73.2% were men. The median age was 65 years (IQR [57.0; 71.0]).

The patients were divided into three sex-specific tertiles based on their CAC scores. Compared to the lowest tertile (CAC score ranging between 0 and 2), patients in the highest tertile (CAC score ranging between 7 and 12) were older (median 60.0, IQR [51.0–66.0] vs. median 69.5, [65.0–74.8]; *p* < 0.001) (Table 1); had a higher prevalence of coronary artery disease (1.1% vs. 17.5%, *p* < 0.001) and diabetes mellitus (7.5% vs. 34.5%, *p* < 0.001), and had a higher ICU mortality (32.3% vs. 62.1%, *p* = 0.001).

**Table 1:** Baseline characteristics of the study population across coronary artery calcification (CAC) tertiles

	Tertile 1 (*N* = 93)	Tertile 2 (*N* = 54)	Tertile 3 (*N* = 58)	*p*-value
Age (years)	60.0 [51.0; 66.0]	67.0 [61.3; 71.8]	69.5 [65.0; 74.8]	< 0.001
BMI (kg/m^2^)	27.8 [25.2; 31.2]	27.7 [24.9; 31.1]	27.7 [24.7; 30.7]	0.939
Gender (men, *N*, %)*	59 (63.4)	43 (79.6)	48 (82.8)	0.015
Smoking (*N*, %)**	6 (6.5)	6 (11.3)	4 (6.9)	0.561
Arrhythmia (*N*, %)***	5 (5.4)	5 (9.4)	11 (19.3)	0.030
Heart failure (*N*, %)***	0 (0.0)	2 (3.8)	3 (5.3)	0.068
Coronary artery disease (*N*, %)***	1 (1.1)	5 (9.4)	10 (17.5)	< 0.001
Myocardial infarction (*N*, %)***	0 (0.0)	3 (5.7)	20 (35.1)	< 0.001
Valvular disease (*N*, %)***	1 (1.1)	0 (0.0)	0 (0.0)	1.000
Diabetes mellitus (*N*, %)	7 (7.5)	11 (20.4)	20 (34.5)	< 0.001
Chronic pulmonary disease (*N*, %)	9 (9.7)	8 (14.8)	10 (17.2)	0.374
Chronic kidney disease (*N*, %)	1 (1.1)	0 (0.0)	7 (12.1)	0.001
APACHE-II (points)	15.0 [13.0; 17.0]	14.5 [13.0; 18.0]	16.0 [14.0; 19.0]	0.004
ICU-mortality (*N*, %)*	30 (32.3)	21 (38.9)	36 (62.1)	0.001
QRST area (µVs)	58.0 [46.1; 79.5]	60.7 [40.1; 80.1]	65.8 [47.5; 82.1]	0.429
QRS area (µVs)	25.9 [19.4; 34.5]	27.5 [19.3; 34.4]	32.2 [26.3; 41.2]	0.006
T area (µVs)	31.0 [23.5; 50.0]	31.6 [16.8; 44.8]	30.5 [18.1; 45.1]	0.529
QRS duration (ms)	99.1 [86.3; 112.7]	101.4 [91.0; 119.4]	110.3 [94.8; 130.4]	0.011
QRS amplitude (mV)	1.0 [0.8; 1.3]	1.1 [0.8; 1.3]	1.1 [0.9; 1.4]	0.296
QTc time (ms)	408.0 (68.2)	415.7 (79.0)	427.7 (82.6)	0.298
Hs-cTnT (ng/L)	11.5 [7.0; 25.3]	14.0 [10.0; 28.0]	20.0 [12.0; 51.8]	0.002
NT-proBNP (pmol/L) (IQR)	47.6 [25.9; 122.8]	67.8 [21.0; 157.0]	92.7 [30.3; 252.8]	0.033

Data are presented as means (standard deviation: ± SD), median [Q1,Q3], or counts (%). Differences were tested using the Kruskal–Wallis test, one way ANOVA or Fisher’s exact test unless indicated otherwise. *APACHE-II* Acute Physiology And Chronic Health Evaluation score, *BMI* body mass index, *hs-cTnT* high-sensitivity troponin-T, *ICU* Intensive Care Unit; *NT-proBNP* N-terminal pro-B-type natriuretic peptide. *X^2^ instead of Fisher exact test;**data missing, *n* = 1;***data missing, *n* = 2

### Associations between CAC tertiles and longitudinal vectorcardiography

**Fig. 2:**
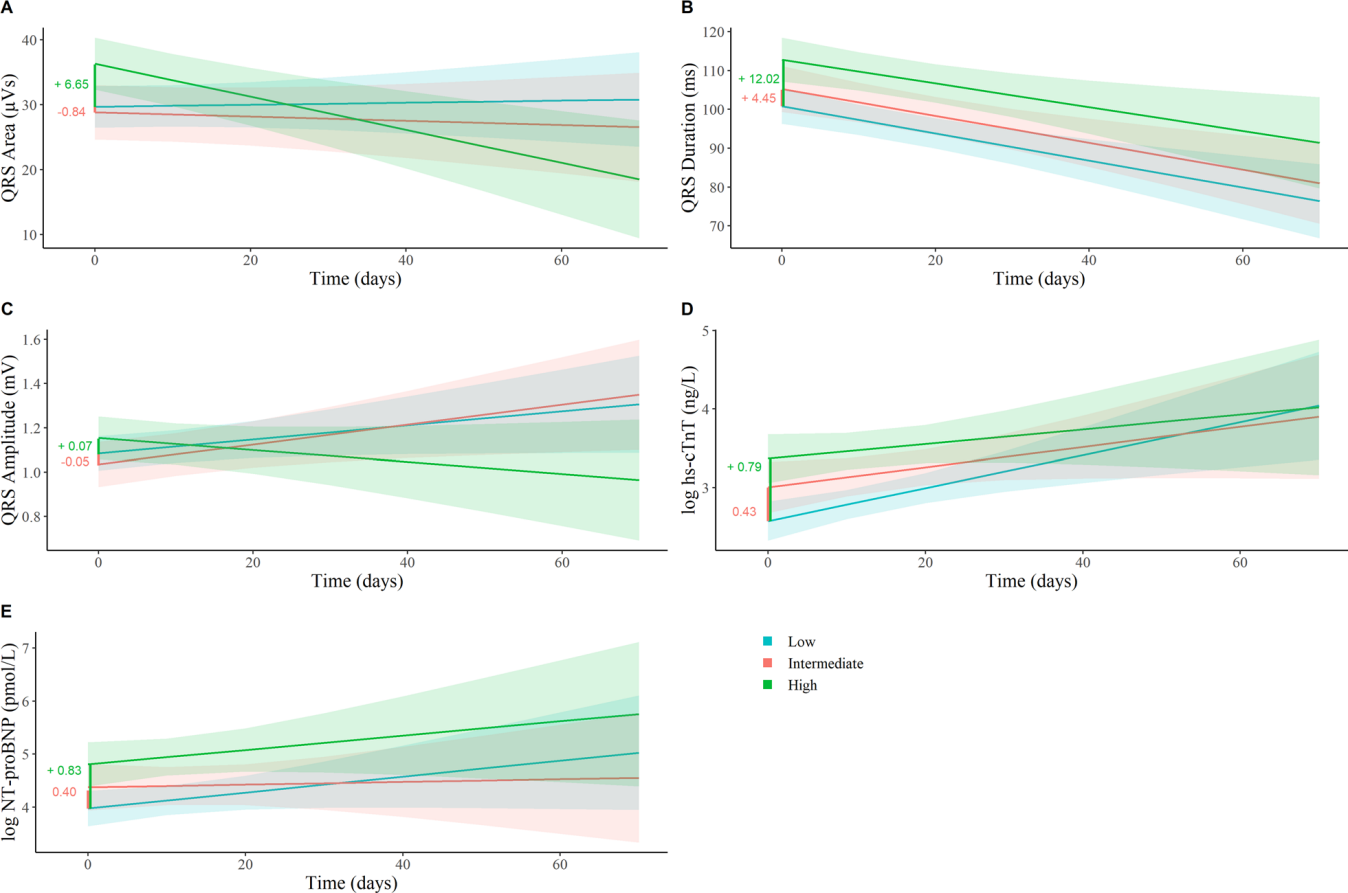
Crude models of QRS Area (**A**), QRS Duration (**B**), QRS Amplitude (**C**), high-sensitivity troponin-T (hs-cTnT) (**D**) and N-terminal pro-B-type natriuretic peptide (NT-proBNP) **E** over time for tertiles of coronary artery calcification (CAC), with 95% confidence intervals and average differences between tertiles (red and green)

In the highest CAC tertile, the QRS area at baseline was 6.65 µVs larger compared to the lowest CAC tertile ([1.50; 11.81], *p* = 0.012), which decreased during admission (-0.27 µVs per day [-0.43;-0.11], *p* = 0.001) (Table 2, model 1; Fig. 2 panel A). After adjustment for age, sex, and APACHE-II score (8.50 µVs [2.69; 14.30], *p* = 0.004), chronic comorbidities (8.26 µVs [2.46; 14.05], *p* = 0.006), and additionally for serum markers (7.59 µVs [2.41; 12.77], *p* = 0.004) the difference remained (Table 2, model 2 and 3; Additional file 1: Table S1, model 4) [9, 28, 30–35].

**Table 2:** Results of linear mixed-effects models: association between coronary artery calcification tertiles and vectorcardiography variables

	QRS-Area (µVs)	QRS Duration (ms)	QRS Amplitude (mV)
	*β* (95%CI); *p*-value	*β* (95%CI); *p*-value	*β* (95%CI); *p*-value
*Model 1: Crude*			
Tertile 1 (reference)	–	–	–
Average difference tertile 2	− 0.84 (− 6.13; 4.44); 0.753	4.45 (− 3.02; 11.93); 0.242	− 0.05 (− 0.18; 0.08); 0.447
Interaction tertile 2 and time (days)	− 0.05 (− 0.20; 0.10); 0.527	0.00 (− 0.24; 0.24); 0.982	0.00 (− 0.00; 0.01); 0.601
Average difference tertile 3	6.65 (1.50; 11.81); 0.012*	12.02 (4.74; 19.30); 0.001*	0.07 (− 0.05; 0.19); 0.271
Interaction tertile 3 and time (days)	− 0.27 (− 0.43; − 0.11); 0.001*	0.04 (− 0.21; 0.29); 0.738	− 0.01 (− 0.01; − 0.00); 0.029*
*Model 2: Model 1 adjusted for age, sex, and APACHE − II score*			
Tertile 1 (reference)	–	–	–
Average difference tertile 2	0.56 (− 5.03; 6.15); 0.844	− 0.45 (− 7.87; 6.96); 0.904	0.02 (− 0.11; 0.15); 0.777
Interaction tertile 2 and time (days)	− 0.05 (− 0.20; 0.10); 0.533	0.00 (− 0.23; 0.24); 0.985	0.00 (− 0.00; 0.01); 0.563
Average difference tertile 3	8.50 (2.69; 14.30); 0.004*	6.07 (− 1.53; 13.67); 0.118	0.16 (0.02; 0.30); 0.023*
Interaction tertile 3 and time (days)	− 0.27 (− 0.43; − 0.11); 0.001*	0.04 (− 0.21; 0.29); 0.772	− 0.01 (− 0.01; − 0.00); 0.038*
*Model 3: Model 2 adjusted for chronic lung disease and liver conditions*			
Tertile 1 (reference)	–	–	–
Average difference tertile 2	0.55 (− 5.10; 6.19); 0.849	− 0.30 (− 7.78; 7.18); 0.937	0.02 (− 0.11; 0.16); 0.717
Interaction tertile 2 and time (days)	− 0.05 (− 0.20; 0.10); 0.533	0.00 (− 0.24; 0.24); 1.000	0.00 (− 0.00; 0.01); 0.542
Average difference tertile 3	8.26 (2.46; 14.05); 0.006*	6.31 (− 1.31; 13.93); 0.105	0.16 (− 0.02; 0.29); 0.025*
Interaction tertile 3 and time (days)	− 0.27 (− 0.43; − 0.11); 0.001*	0.03 (− 0.22; 0.28); 0.804	− 0.01 (− 0.01; 0.00); 0.040*

Regression coefficients (*β*) indicate the average difference of the variable under study between CAC tertiles, with tertile 1, the lowest CAC, as reference. The interaction between a tertile with time indicates the average increase or decrease over time. *APACHE-II* Acute Physiology And Chronic Health Evaluation score.*p-value < 0.05 and a *p*-value for interaction < 0.10

Patients in the highest CAC tertile had the longest QRS duration (12.02 ms longer [4.74; 19.30], *p* = 0.001) (Table 2, model 1; Fig. 2 panel B), compared to the lowest CAC tertile. Moreover, these patients had a 0.07 mV higher ([-0.05; 0.19], *p* = 0.271) QRS amplitude compared to the lowest CAC tertile, although this result was not statistically significant (Table 2, model 1; Fig. 2 panel C). After adjustment for age, sex, and APACHE-II score, patients in the highest CAC tertile had a higher QRS amplitude (0.16 mV higher [0.02; 0.30], p = 0.023) compared to the lowest tertile and the QRS amplitude decreased during admission (− 0.01 mV/per day [− 0.01; − 0.00], *p* = 0.038 (Table 2, model 2).

### Additional analyses CAC-scores

When data were re-analyzed using CAC as a continuous variable, QRS area and QRS amplitude showed similar patterns over time compared to the tertiles analysis (Additional file 1: Table S2, model 1). After adjustment for age, sex, and APACHE-II score, the analyses showed that per 1 unit increase in CAC-scores, the QRS area was 0.840 µVs ([0.138; 1.541], *p* = 0.019) larger on average over time and decreased by − 0.026 µVs [− 0.044; − 0.007] (*p* = 0.007) (Additional file 1: Table S1, model 2). These results remained similar after additional adjustments for chronic lung disease and liver conditions (Additional file 1: Table S3, model 3).

### Serum markers

In the highest CAC tertile, log hs-cTnT (0.79 ng/L higher [0.40; 1.19], *p* < 0.001) and log NT-proBNP (0.83 pmol/L higher [0.30; 1.37], *p* = 0.002) were on average higher over time compared to the lowest and intermediate tertile (Table 3, model 1). Log hs-cTnT remained highest in the highest CAC tertile after adjustment for age, sex, APACHE-II score (model 2) (0.37 ng/L higher [0.01; 0.74], *p* = 0.046) and chronic lung disease, and liver conditions (model 3) (0.44 ng/L higher [0.04; 0.83], *p* = 0.031). These adjustments attenuated the associations between CAC and log NT-proBNP. Log hs-cTnT and log NT-proBNP did not show a statistically significant increase or decrease during admission in the highest and intermediate tertile, compared to the lowest CAC-tertile (Table 3, model 1).

**Table 3:** Results of linear mixed-effects models: association between coronary artery calcification tertiles and serum biomarkers

	Log hs-cTNT (ng/L)	Log NT-proBNP (pmol/L)
	*β* (95%CI); *p*-value	*β* (95%CI); *p*-value
*Model 1: Crude*		
Tertile 1 (reference)	–	–
Average difference tertile 2	0.43 (0.02; 0.84); 0.042	0.40 (-0.16; 0.95); 0.157
Interaction tertile 2 and time	− 0.01 (− 0.03; 0.01); 0.392	− 0.01 (− 0.04; 0.02); 0.366
Average difference tertile 3	0.79 (0.40; 1.19); < 0.001*	0.83 (0.30; 1.37); 0.002*
Interaction tertile 3 and time	− 0.01 (− 0.03; 0.01); 0.233	− 0.00 (− 0.03; 0.03); 0.917
*Model 2: Model 1 adjusted for age, sex, and APACHE-II score*		
Tertile 1 (reference)	–	–
Average difference tertile 2	0.22 (− 0.14; 0.58); 0.233	0.27 (− 0.30; 0.84); 0.348
Interaction tertile 2 and time	− 0.01 (− 0.02; 0.01); 0.300	− 0.01 (− 0.03; 0.02); 0.543
Average difference tertile 3	0.37 (0.01; 0.74); 0.046*	0.39 (− 0.17; 0.95); 0.176
Interaction tertile 3 and time	− 0.01 (− 0.02; 0.01); 0.389	0.01 (− 0.01; 0.04); 0.376
*Model 3: Model 2 adjusted for chronic lung disease and liver conditions*		
Tertile 1 (reference)	–	–
Average difference tertile 2	0.25 (− 0.14; 0.65); 0.206	0.31 (− 0.26; 0.88); 0.289
Interaction tertile 2 and time	− 0.01 (− 0.03; 0.01); 0.361	− 0.01 (− 0.04; 0.02); 0.499
Average difference tertile 3	0.44 (0.04; 0.83); 0.031*	0.43 (− 0.13; 1.00); 0.133
Interaction tertile 3 and time	− 0.01 (− 0.03; 0.01); 0.270	0.01 (− 0.02; 0.04); 0.437

Regression coefficients (*β*) indicate the average difference of the variable under study between CAC tertiles, with tertile 1, the lowest CAC, as reference. The interaction between a tertile with time indicates the average increase or decrease over time. *APACHE-II* Acute Physiology And Chronic Health Evaluation score, *hs-cTnT* high-sensitivity troponin-T, *NT-proBNP* N-terminal pro-B-type natriuretic peptide. *p-value < 0.05 and a *p*-value for interaction < 0.10

## Discussion

This study examined whether a high degree of CAC is associated with VCG-derived variables in mechanically ventilated COVID-19 patients over time. This study has four main findings. First, patients with the highest degree of CAC had the largest QRS area at baseline, as opposed to those with less or no CAC. At baseline, QRS duration was longer in patients with the highest degree of CAC. The QRS area, consisting of QRS duration and amplitude, decreased significantly over time. Second, this association was mainly driven by a higher QRS amplitude, which decreased during admission and not by QRS duration, indicating loss of myocardial tissue. Third, these results were independent of serum markers and potential confounders, including age, sex, APACHE-II score, chronic lung disease, and/or liver conditions. Lastly, patients with the highest degree of CAC had higher serum cardiac biomarkers over time, including hs-cTnT and NT-proBNP, indicating myocardial injury.

VCG is a technique that records the electrical forces of the heart in three directions and is a more detailed and standardized alternative for ECG measurements. This is important as daily ECG assessment in mechanically ventilated COVID-19 patients showed widespread ECG abnormalities reflective of conduction abnormalities, including RV strain characteristics, *P*-wave splitting, QRS fragmentations, and changes reflective of myocardial ischemia/inflammation, including ST-segment deviations and flat *T*-waves. However, no differences were found between survivors and non-survivors based on ECG [3]. Although somewhat more complex than the ECG, VCG calculates the QRS area and amplitude more objectively [36] and has been proven to achieve a higher sensitivity for detecting ischemic heart disease compared to the ECG [37]. Therefore, VCG is likely to be more precise when investigating trajectories of cardiac electrical conduction and loss of myocardial tissue compared to ECG.

Previously, it was shown that a high degree of CAC is associated with more severe organ failure and mortality in mechanically ventilated COVID-19 patients [9, 11]. Moreover, other studies showed that a high degree of CAC was associated with more myocardial stress in COVID-19 patients [12]. Regarding the association between CAC and ECG patterns, studies showed that CAC was associated with ECG-derived QT interval and QRS duration[38]. By using VCG-derived variables, the present investigation extends previous evidence with mixed results. Previous studies have shown that VCG-derived QRS area is inversely related to focal scarring on cardiac magnetic resonance imaging (CMR) [39]. The QRS score is composed of different criteria that can be used to estimate the degree of myocardial scar [40–42]. There is evidence demonstrating that ECG derived QRS scores can identify scar tissue in patients with ischemic and nonischemic cardiomyopathy [40]. The current results suggest that a higher degree of CAC, reflecting more atherosclerosis, might contribute to a loss of myocardial tissue in critically ill patients. However, as myocardial tissue was not visualized in the current study, future research utilizing imaging modalities, such as CMR or echocardiography, could provide further insights. Moreover, the degree of stenosis due to atherosclerosis, using the coronary artery disease-reporting and data system (CAD-RADS) could provide further insights [43].

In addition to VCG, the association between CAC and biomarkers including hs-cTnT and NT-proBNP was investigated. Hs-cTnT is a highly sensitive cardiac injury biomarker that has been associated with myocardial injury [44, 45]. NT-proBNP is a reliable biomarker for detecting myocardial wall stress [46]. In previous studies, serum markers including hs-cTnT and NT-proBNP have been found to be associated with coronary artery disease and CAC [47–50]. Furthermore, in COVID-19 patients, these serum markers were associated with mortality [3, 5–8]. The current results show that hs-cTnT and NT-proBNP levels were higher in patients with the highest degree of CAC. These results are in line with those of previous studies, and suggest myocardial stress in these patients.

This study has several strengths. First, confounding was extensively addressed by using adjusted models in all analyses. Next, CAC was assessed by 2 experienced readers using a semi quantitative system. Furthermore, the serial data design showed that small differences in trajectories per day could be detected, proving that the analysis was precise.

However, the study included only COVID-19 patients within one center, limiting the generalizability of the results. Other critical care variables might have influenced the ECGs and VCGs, such as pulmonary edema. Due to the observational design of our study, residual confounding can not be exluded, although we dealt with confounding extensively. Furthermore, cut-off points on the VCG variables were not investigated and therefore not defined in this study. Nevertheless, the association of decreasing QRS amplitude over time in patients with more CAC could still be a helpful monitoring variable in the ICU as an early detector of myocardial function loss, aiding the decision to employ clinical imaging modalities such as echocardiography.

## Conclusions

In conclusion, mechanically ventilated COVID-19 patients with a high degree of CAC had a larger QRS area, which consist of QRS amplitude and duration. QRS area and QRS amplitude decreased during admission, which suggests that CAC might contribute to a decrease in myocardial tissue. This study enhances our understanding of CAC and trajectories of VCG-variables and suggests different patterns of electrical conduction over time during critical illness. These insights could improve risk stratification and prognostication of patients with critical illness. More research utilizing imaging modalities, such as CMR or echocardiography, could provide further insights.

### Supplementary Information

**Additional file 1.** Supplementary tables.

## Data Availability

The datasets generated and analysed during the current study are not publicly available, but are available from the corresponding author on reasonable request and can only be shared within legal boundaries.

## Abbreviations

APACHE-II: Acute Physiology and Chronic Health Evaluation score
BMI: Body mass index
CAC: Coronary artery calcification
CK-MB: Creatine kinase-MB
CO-RADS: COVID-19 Reporting and Data System
COVID-19: Coronavirus-19
CT: Computed tomography
ECG: Electrocardiogram
Hs-cTnT: High-sensitivity troponin-T
ICU: Intensive Care Unit
IQR: Interquartile range
MaastrICCht: Maastricht Intensive Care COVID
MUMC +: Maastricht University Medical Center +
NT-proBNP: N-terminal pro-B-type natriuretic peptide
PCR: Polymerase chain reaction
SARS-CoV-2: Severe acute respiratory syndrome coronavirus 2
VCG: Vectorcardiography
