# Supplementary material for: The association between coronary artery calcification and vectorcardiography in mechanically ventilated COVID-19 patients: the Maastricht Intensive Care COVID cohort
Source: Intensive Care Med Exp. 2024 Mar 7;12:26. doi: 10.1186/s40635-024-00611-0 (PMC10920503; doi:10.1186/s40635-024-00611-0)
Supplement: Supplementary file 1 — Additional file 1. Supplementary tables. [file 40635_2024_611_MOESM1_ESM.docx]

**Additional file 1**

**Table S1:** Additional results of linear mixed-effects models: association between coronary artery calcification (CAC)-tertiles and vectorcardiography (VCG) variables over time adjusted for high-sensitivity troponin-T (hs-cTnT) and N-terminal pro-B-type natriuretic peptide (NT-proBNP)

|  |  | ***QRS-Area (µVs)*** | ***QRS Duration (ms)*** | | ***QRS Amplitude (mV)*** |
| --- | --- | --- | --- | --- | --- |
|  |  | β (95%CI); p-value | β (95%CI); p-value | | β (95%CI); p-value |
| ***Model 1:*** *Crude* | |  |  |  | |
|  | Tertile 1 (reference) | - | - | | - |
|  | Average difference tertile 2 | -0.84 (-6.13; 4.44); 0.753 | 4.45 (-3.02; 11.93); 0.242 | | -0.05 (-0.18; 0.08); 0.447 |
|  | Interaction tertile 2 and time (days) | -0.05 (-0.20; 0.10); 0.527 | 0.00 (-0.24; 0.24); 0.982 | | 0.00 (-0.00; 0.01); 0.601 |
|  | Average difference tertile 3 | 6.65 (1.50; 11.81); 0.012* | 12.02 (4.74; 19.30); 0.001* | | 0.07 (-0.05; 0.19); 0.271 |
|  | Interaction tertile 3 and time (days) | -0.27 (-0.43;-0.11); 0.001* | 0.04 (-0.21; 0.29); 0.738 | | -0.01 (-0.01; -0.00); 0.029* |
| ***Model 2:*** *Model 1 adjusted for NT-proBNP (pmol/L)* | | | | | |
|  | Tertile 1 (reference) | - | - | | - |
|  | Average difference tertile 2 | -0.85 (-6.14 ; 4.43 ); 0.752 | 4.45 (-3.02 ; 11.92 ); 0.242 | | -0.05 (-0.18 ; 0.08 ); 0.446 |
|  | Interaction tertile 2 and time (days) | -0.05 (-0.20 ; 0.10 ); 0.530 | 0.00 (-0.23 ; 0.24 ); 0.984 | | 0.00 (0.00 ; 0.01); 0.601 |
|  | Average difference tertile 3 | 6.55 (1.39 ; 11.71 ); 0.013* | 12.08 (4.81 ; 19.36 ); 0.001* | | 0.07 (-0.06 ; 0.19 ); 0.278 |
|  | Interaction tertile 3 and time (days) | -0.27 (-0.43 ; -0.11 ); 0.001* | 0.04 (-0.21 ; 0.29 ); 0.740 | | -0.01 (-0.01 ; 0.00); 0.030* |
| ***Model 3:*** *Model 1 adjusted for hs-cTNT (ng/L)* | | | | | |
|  | Tertile 1 (reference) | - | - | |  |
|  | Average difference tertile 2 | -0.79 (-6.08 ; 4.49 ); 0.768 | 4.61 (-2.86 ; 12.08 ); 0.226 | | -0.05 (-0.17 ; 0.08); 0.484 |
|  | Interaction tertile 2 and time (days) | -0.05 (-0.20 ; 0.10 ); 0.499 | -0.01 (-0.25 ; 0.23 ); 0.956 | | 0.00 (0.00 ; 0.01); 0.673 |
|  | Average difference tertile 3 | 6.73 ( 1.58 ; 11.89 ); 0.011* | 12.25 (4.97 ; 19.52 ); 0.001* | | 0.08 (-0.05 ; 0.20); 0.236 |
|  | Interaction tertile 3 and time (days) | -0.27 (-0.43 ; -0.11 ); 0.001* | 0.04 (-0.21 ; 0.29 ); 0.762 | | -0.01 (-0.01 ; 0.00); 0.026* |
| ***Model 4:*** *Model 1 adjusted for NT-proBNP (pmol/L) and hs-cTNT (ng/L)* | | | | | |
|  | Tertile 1 (reference) | - | - | | - |
|  | Average difference tertile 2 | -0.79 (-6.07 ; 4.49 ); 0.769 | 4.61 (-2.85 ; 12.07 ); 0.225 | | -0.05 (-0.17 ; 0.08); 0.485 |
|  | Interaction tertile 2 and time (days) | -0.05 (-0.20 ; 0.10 ); 0.496 | -0.01 (-0.25 ; 0.23 ); 0.955 | | 0.00 (0.00 ; 0.01); 0.676 |
|  | Average difference tertile 3 | 6.63 ( 1.48 ; 11.79 ); 0.012* | 12.30 (5.03 ; 19.57 ); 0.001* | | 0.07 (-0.05 ; 0.20); 0.244 |
|  | Interaction tertile 3 and time (days) | -0.27 (-0.43 ; -0.11 ); 0.001* | 0.04 (-0.21 ; 0.29 ); 0.773 | | -0.01 (-0.01 ; 0.00); 0.027* |

Regression coefficients (β) indicate the average difference of the variable under study between CAC tertiles, with tertile 1, the lowest CAC, as reference. The interaction between a tertile with time indicates the average increase or decrease over time. * p-value <0.05 or a p-value for interaction <0.10. Hs-cTnT, high-sensitivity troponin-T; NT-proBNP, N-terminal pro-B-type natriuretic peptide

**Table S2:** Additional results of linear mixed-effects models: association between coronary artery calcification (CAC)-tertiles and vectorcardiography (VCG) variables over time adjusted for age, sex, Acute Physiology And Chronic Health Evaluation score (APACHE-II) and comorbidities

|  |  | ***QRS T-Area (µVs)*** | ***T-Area (µVs)*** | | ***QTc-Time (ms)*** |
| --- | --- | --- | --- | --- | --- |
|  |  | β (95%CI); p-value | β (95%CI); p-value | | β (95%CI); p-value |
| ***Model 1:*** *Crude* | |  |  |  | |
|  | Tertile 1 (reference) | - | - | | - |
|  | Average difference tertile 2 (t=0) | -6.80 (-17.54; 3.94); 0.214 | -6.11 (-12.98; 0.76); 0.082 | | 5.29 (-7.81; 18.40); 0.428 |
|  | Interaction tertile 2 and time (days) | 0.14 (-0.29; 0.58); 0.520 | 0.20 (-0.14; 0.54); 0.257 | | 0.51 (-0.14; 1.16); 0.125 |
|  | Average difference tertile 3 (t=0) | 0.82 (-9.62; 11.25); 0.878 | -5.87 (-12.52; 0.77); 0.083 | | 9.55 (-3.09; 22.19); 0.139 |
|  | Interaction tertile 3 and time (days) | -0.22 (-0.68; 0.23); 0.336 | 0.07 (-0.28; 0.43); 0.683 | | 0.86 (0.17; 1.54); 0.015* |
| ***Model 2:*** *Model 1 adjusted for age, sex, and APACHE-II score* | | | | | |
|  | Tertile 1 (reference) | - | - | | - |
|  | Average difference tertile 2 | -4.84 (-16.02; 6.34); 0.395 | -5.88 (-12.95; 1.20); 0.104 | | 2.24 (-11.38; 15.85); 0.747 |
|  | Interaction tertile 2 and time (days) | 0.14 (-0.30; 0.58); 0.520 | 0.21 (-0.13; 0.56); 0.229 | | 0.50 (-0.15; 1.15); 0.130 |
|  | Average difference tertile 3 | 3.94 (-7.45; 15.33); 0.497 | -4.68 (-11.77; 2.40); 0.195 | | 6.14 (-7.63; 19.91); 0.381 |
|  | Interaction tertile 3 and time (days) | -0.23 (-0.68; 0.23); 0.332 | 0.08 (-0.28; 0.44); 0.675 | | 0.83 (0.15; 1.52); 0.018* |
| ***Model 3:*** *Model 2 adjusted for chronic lung disease and liver conditions* | | | | | |
|  | Tertile 1 (reference) | - | - | | - |
|  | Average difference tertile 2 | -5.56 (-16.77; 5.65); 0.331 | -6.63 (-13.73; 0.46); 0.067 | | 3.59 (-10.15; 17.32); 0.608 |
|  | Interaction tertile 2 and time (days) | 0.15 (-0.29; 0.59); 0.507 | 0.21 (-0.13; 0.55); 0.232 | | 0.51 (-0.15; 1.16); 0.129 |
|  | Average difference tertile 3 | 3.14 (-8.23; 14.51); 0.587 | -5.30 (-12.38; 1.79); 0.143 | | 6.94 (-6.85; 20.74); 0.324 |
|  | Interaction tertile 3 and time (days) | -0.21 (-0.66; 0.25); 0.378 | 0.10 (-0.26; 0.45); 0.589 | | 0.82 (0.13; 1.51); 0.021* |

Regression coefficients (β) indicate the average difference of the variable under study between CAC tertiles, with tertile 1, the lowest CAC, as reference. The interaction between a tertile with time indicates the average increase or decrease over time. * p-value <0.05 or a p-value for interaction <0.10. APACHE-II, Acute Physiology And Chronic Health Evaluation score

**Table S3:** Results of linear mixed-effects models: association between coronary artery calcification (CAC)-scores as continuous variable and vectorcardiography (VCG) variables over time adjusted for age, sex, Acute Physiology And Chronic Health Evaluation score (APACHE-II) and comorbidities

|  |  | ***QRST area (µVs)*** | ***QRS area (µVs)*** | ***T area (µVs)*** | ***QRS duration (ms)*** | ***QRS amplitude (mV)*** | ***QTc time (ms)*** |
| --- | --- | --- | --- | --- | --- | --- | --- |
|  |  | β (95%CI);  p-value | β (95%CI);  p-value | β (95%CI);  p-value | β (95%CI);  p-value | β (95%CI);  p-value | β (95%CI);  p-value |
| ***Model 1:*** *Crude* | |  |  |  |  |  |  |
|  | CAC-score | -0.223 (-1.436; 0.990); 0.718 | 0.591 (-0.013; 1.195); 0.055 | -0.808 (-1.571; -0.044); 0.039* | 1.534 (0.700; 2.369); <0.001* | 0.004 (-0.010; 0.019); 0.551 | 1.572 (0.121; 3.023); 0.034* |
|  | Interaction CAC score and time | -0.013 (-0.065; 0.038); 0.611 | -0.026 (-0.045; -0.008); 0.006* | 0.013 (-0.026; 0.053); 0.507 | 0.005 (-0.024; 0.033); 0.746 | -0.001 (-0.001; 0.000); 0.051* | 0.078 (0.001; 0.155); 0.047* |
| ***Model 2:*** *Model 1 adjusted for age, sex, and APACHE-II score* | | | | |  |  |  |
|  | CAC-score | 0.164 (-1.189; 1.517); 0.812 | 0.840 (0.138; 1.541); 0.019* | -0.670 (-1.502; 0.163); 0.115 | 0.837 (-0.064; 1.738); 0.689 | 0.016 (-0.000; 0.032); 0.054 | 1.221 (-0.399; 2.842); 0.140 |
|  | Interaction CAC score and time | -0.014 (-0.065; 0.038); 0.609 | -0.026 (-0.044; -0.007); 0.007* | 0.013 (-0.027; 0.054); 0.521 | 0.005 (-0.023; 0.034); 0.715 | -0.001 (-0.001; 0.000); 0.061* | 0.076 (-0.001; 0.153); 0.052* |
| ***Model 3:*** *Model 2 adjusted for chronic lung disease and liver conditions* | | | | |  |  |  |
|  | CAC-score | 0.078 (-1.277; 1.434); 0.910 | 0.827 (0.128; 1.526); 0.021* | -0.753 (-1.586; 0.081); 0.077 | 0.876 (-0.028; 1.781); 0.058 | 0.016 (-0.000; 0.032); 0.054 | 1.342 (-0.283; 2.966); 0.106 |
|  | Interaction CAC score and time | -0.012 (-0.064; 0.040); 0.649 | -0.026 (-0.044; -0.007); 0.006* | 0.016 (-0.024; 0.056); 0.445 | 0.005 (-0.024; 0.033); 0.747 | -0.001 (-0.001; 0.000); 0.063* | 0.074 (-0.003; 0.152); 0.060* |

Regression coefficients (β) indicate the average difference per 1 unit in CAC-scores of the variable under study. The interaction between CAC-score and time indicates the average increase or decrease over time. *p<0.05 or a p-value for interaction <0.10. APACHE-II, Acute Physiology And Chronic Health Evaluation score

**Table Sa4:** Results of linear mixed-effects models: association between coronary artery calcification tertiles and serum biomarkers, backtransformed from log-values

|  |  | ***Hs-cTNT (ng/L)*** | ***NT-proBNP (pmol/L)*** |  |  |
| --- | --- | --- | --- | --- | --- |
|  |  | β (95%CI); p-value | β (95%CI); p-value |  |  |
| ***Model 1:*** *Crude* | |  |  | | |
|  | Tertile 1 (reference) | - | - |  |  |
|  | Average difference tertile 2 | 1.54 (1.02 ;2.32); 0.042 | 1.49 (0.86; 2.60); 0.157 |  |  |
|  | Interaction tertile 2 and time | 0.99 (0.97 ;1.01); 0.392 | 0.99 (0.96; 1.01); 0.366 |  |  |
|  | Average difference tertile 3 | 2.21 (1.48 ;3.30); <0.001* | 2.30 (1.35; 3.93); 0.002* |  |  |
|  | Interaction tertile 3 and time | 0.99 (0.97;1.01); 0.233 | 1.00 (0.97; 1.03); 0.917 |  |  |
| **Model 2:** Model 1 adjusted for age, sex, and APACHE-II score | | | | |  |
|  | Tertile 1 (reference) | - | - |  |  |
|  | Average difference tertile 2 | 1.25 (0.87; 1.79); 0.233 | 1.31 (0.74; 2.32 ); 0.348 |  |  |
|  | Interaction tertile 2 and time | 0.99 (0.98; 1.01); 0.300 | 0.99 (0.97; 1.02 ); 0.542 |  |  |
|  | Average difference tertile 3 | 1.45 (1.01; 2.09); 0.046* | 1.47 (0.84; 2.59 ); 0.176 |  |  |
|  | Interaction tertile 3 and time | 0.99 (0.98; 1.01); 0.389 | 1.01 (0.99; 1.04 ); 0.376 |  |  |
| **Model 3:** Model 2 adjusted for chronic lung disease and liver conditions | | | | |  |
|  | Tertile 1 (reference) | - | - |  |  |
|  | Average difference tertile 2 | 1.29 (0.87; 1.91); 0.206 | 1.36 (0.77; 2.41); 0.289 |  |  |
|  | Interaction tertile 2 and time | 0.99 (0.97; 1.01); 0.361 | 0.99 (0.97; 1.02); 0.499 |  |  |
|  | Average difference tertile 3 | 1.55 (1.04; 2.30); 0.031* | 1.54 (0.88; 2.71); 0.133 |  |  |
|  | Interaction tertile 3 and time | 0.99 (0.97; 1.01); 0.270 | 1.01 (0.98; 1.04); 0.437 |  |  |

Regression coefficients (β) indicate the average difference of the variable under study between CAC tertiles, with tertile 1, the lowest CAC, as reference. The interaction between a tertile with time indicates the average increase or decrease over time. **Abbreviations:** APACHE-II, Acute Physiology And Chronic Health Evaluation score; hs-cTnT, high-sensitivity troponin-T; NT-proBNP, N-terminal pro-B-type natriuretic peptide. * p-value <0.05 and a p-value for interaction <0.10.
